# Localizing Tungsten Single Atoms around Tungsten Nitride Nanoparticles for Efficient Oxygen Reduction Electrocatalysis in Metal–Air Batteries

**DOI:** 10.1002/advs.202105192

**Published:** 2022-06-22

**Authors:** Yuanyuan Ma, Yong Yu, Junhui Wang, Jason Lipton, Hui Ning Tan, Lirong Zheng, Tong Yang, Zhaolin Liu, Xian Jun Loh, Stephen J. Pennycook, Lei Shen, Zongkui Kou, André D. Taylor, John Wang

**Affiliations:** ^1^ Department of Materials Science and Engineering Faculty of Engineering National University of Singapore Singapore 117574 Singapore; ^2^ Department of Chemical and Biomolecular Engineering Tandon School of Engineering New York University Brooklyn NY 11201 USA; ^3^ Chemical Sciences and Engineering Division Argonne National Laboratory Lemont IL 60439 USA; ^4^ Beijing Synchrotron Radiation Facility Institute of High Energy Physics Chinese Academy of Sciences Beijing 100049 China; ^5^ Department of Applied Physics The Hong Kong Polytechnic University Hung Hom Hong Kong 999077 P. R. China; ^6^ Institute of Materials Research and Engineering Agency for Science Technology and Research (A* STAR) 2 Fusionopolis Way Innovis 138634 Singapore; ^7^ Department of Mechanical Engineering National University of Singapore Singapore 117575 Singapore; ^8^ State Key Laboratory of Advanced Technology for Materials Synthesis and Processing Wuhan University of Technology Wuhan 430070 P. R. China

**Keywords:** metal–air batteries, oxygen reduction reaction, single atom catalysts, synergistic effect, tungsten nitrides

## Abstract

Combining isolated atomic active sites with those in nanoparticles for synergizing complex multistep catalysis is being actively pursued in the design of new electrocatalyst systems. However, these novel systems have been rarely studied due to the challenges with synthesis and analysis. Herein, a synergistically catalytic performance is demonstrated with a 0.89 V (vs reversible hydrogen electrode) onset potential in the four‐step oxygen reduction reaction (ORR) by localizing tungsten single atoms around tungsten nitride nanoparticles confined into nitrogen‐doped carbon (W SAs/WNNC). Through density functional theory calculations, it is shown that each of the active centers in the synergistic entity feature a specific potential‐determining step in their respective reaction pathway that can be merged to optimize the intermediate steps involving scaling relations on individual active centers. Impressively, the W SAs/WNNC as the air cathode in all‐solid‐state Zn‐air and Al‐air batteries demonstrate competitive durability and reversibility, despite the acknowledged low activity of W‐based catalyst toward the ORR.

## Graphical abstract

A unique oversaturation method is employed to synthesize the ensemble catalysts, which comprises the tungsten single atoms and tungsten nitride nanoparticles. The synergistic binary system realizes an efficient four‐step process, oxygen reduction reaction, and gives good electrochemical performance when used as the electrodes for metal–air batteries.

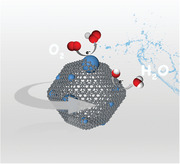

## 1 Introduction

The performance of metal‐air batteries is largely limited by the oxygen reduction reaction (ORR) at the air cathodes. Platinum group (PG) metal electrocatalysts have been known to possess appealing catalytic activity,^[^ ^1^, ^2^, ^3^ ^]^ however, their poor stability and cost have driven considerable efforts to develop alternative transition metal‐based catalysts with excellent catalytic performance. We note that both the catalytic activity and stability are structure‐sensitive properties, which can be optimized by tailoring the active material composition, degree of dispersion, crystallinity, surface area, and surface functional groups.^[^ ^4^, ^5^, ^6^ ^]^ Previously, it has been shown that moving from nanoparticles to single atoms can optimize the metal dispersion, which allows more active species and the exposed surface to participate in catalytic reactions.^[^ ^7^ ^]^ The catalytic activity can be further enhanced by modifying the electronic structure, changing the atomic coordination environment, and adjusting the electron confinement between the metal atoms and the supporting substrate.^[^ ^8^, ^9^, ^10^ ^]^

While nanoparticles and single atoms have been explored for energy storage and conversion applications,^[^ ^1^, ^11^, ^12^, ^13^, ^14^ ^]^ few studies have been made that strategically combine them together. We suggest that combining catalytic active centers with different potential‐determining steps could be an attractive pathway to circumvent the adsorption‐energy scaling relationships on individual active centers.^[^ ^15^, ^16^, ^17^ ^]^ And consequently, this would improve the overall catalytic performance through step coordination to reduce the overall energy barrier in the system. Several recent studies have successfully demonstrated that the combination of single atoms and nanoparticles gives rise to superior catalytic properties compared to either of the individual counterparts in their corresponding reaction.^[^ ^18^, ^19^, ^20^, ^21^ ^]^

Among the various non‐PG metal‐based materials for ORR, tungsten (W)‐based materials, such as tungsten carbide and nitride nanoparticles, have recently attracted considerable interest, owing to their noble‐metal‐like d‐state density in electronic structure around the Fermi level, which enables good oxygen chemisorption.^[^ ^22^, ^23^ ^]^ However, the oxygen‐binding energy of W‐based materials is quite strong, which hinders the competitive adsorption of OH^−^ species on the W atom surface.^[^ ^24^ ^]^ Therefore, applying W‐based catalysts for ORR is still a challenge.

Inspired by the strong synergy among the active centers from more than one catalytic entity, W single atoms coupled with tungsten nitride (W_3_N_4_) nanoparticle ensembles were investigated in this work. Here, we examine the catalytic behavior of ensembled single atoms and nanoparticles in ORR and the relationship between the catalytic activity and the chemical environment. A unique over‐saturation method was first introduced to obtain the combined entity on a nitrogen‐doped carbon substrate through entrapping W‐based polyoxometalate (W‐POM) particles into the inner cavities of zeolitic imidazolate framework‐8 (ZIF‐8), followed by subsequent pyrolysis. During the formation procedure, the ZIF‐8 was used as a sacrificial template, with the cage framework filled by W‐POM. ZIF‐8 prevents metal by confining and separating W‐POM species during catalyst synthesis to form the single atoms. Further, the porous structure of ZIF‐8 perseveres after the pyrolysis process, giving rise to a high surface area of the resulting catalyst. When evaluated as an ORR catalyst, ensembled tungsten single atoms on W_3_N_4_ nanocrystals (W SAs/WNNC) give a high onset potential of 0.89 V versus reversible hydrogen electrode (RHE) and a half‐wave potential of 0.83 V versus RHE. The W SAs/WNNC were then directly applied as an air cathode for both all‐solid‐state Zn–air and Al–air batteries, which demonstrate a durable performance (≈13 h for a Zn‐air battery and 250 min for an Al‐air battery). The present study represents a timely addition to the valuable library of synergistic electrocatalysts for high‐performance metal–air batteries.

## 2 Results and Discussion

### 2.1 Preparation of the Catalysts

**Figure 1:**
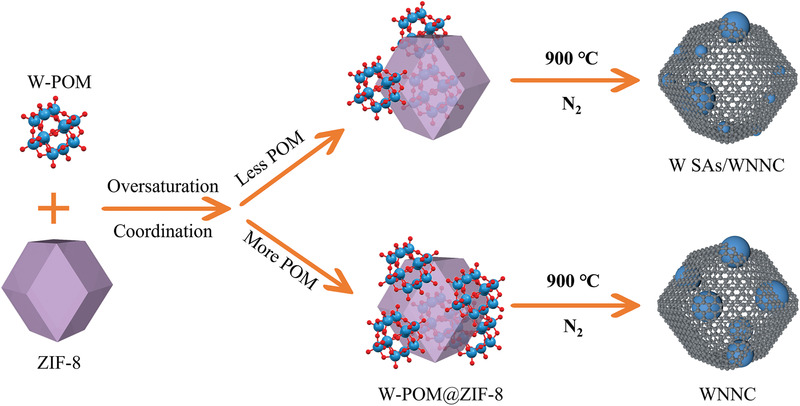
Synthesis routes of W SAs/WNNC and WNNC.

We illustrate the synthesis routes for W SAs/WNNC and WNNC in **Figure** **1**. First, ZIF‐8 is prepared as the confinement substrate, as well as the template for the porous structure. The diameter of ZIF‐8's inner cavity matches well to encase the metal carrier, W‐POM. After the one‐step growth of W‐POM@ZIF‐8, the pyrolysis process renders the conversion of W‐POM@ZIF‐8 into W SAs/WNNC. The oversaturation method is refined from one of our previous spatial confinement methods for single‐atom catalyst synthesis.^[^ ^25^ ^]^ This “one species on one cage” approach allows enhanced size control of the trapped species, and the spatial confinement also restricts the aggregation to obtain high‐loading of single atoms.

By adjusting the precursor loading, a tunable combination of single atoms and nanoparticles can be obtained (Figure 1). In this work, we modified this method, and prepared a series of W‐POM@ZIF‐8 with different amounts of W‐POM added, denoted as W‐POM@ZIF‐8‐*x* (where *x* is the amount of W‐POM, in milligrams, in the following values: 5, 10, 20, 40 mg). The subsequent pyrolytic products were indicated by W SAs/WNNC‐*x* and WNNC‐*x*. The pristine ZIF‐8 and pyrolyzed products (NC) without W components were also synthesized for comparison purposes.

### 2.2 Material Characterizations

**Figure 2:**
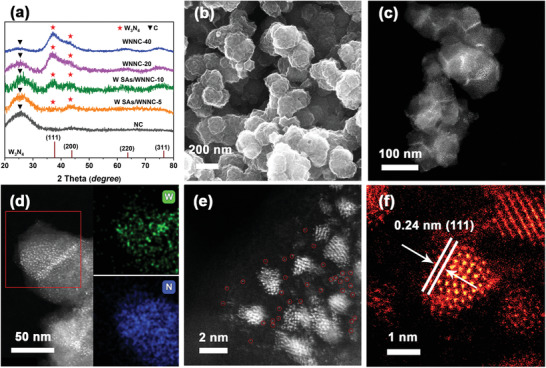
a) XRD curves of all samples. b) SEM images of W SAs/WNNC‐5. c) HAADF‐STEM image of low‐magnification image of W SAs/WNNC‐5. d) STEM‐EDS mapping image showing W and N elements. High‐resolution STEM image of e) W single atom (red circles) ensembled W_3_N_4_ nanoparticle and f) a typical W_3_N_4_ nanoparticle.

The precursors and final catalysts were examined for phases using powder X‐ray diffraction (XRD) (Figure S1, Supporting Information; **Figure** **2**a). The XRD patterns of the W‐POM@ZIF‐8 precursors correspond exactly to that of the standard ZIF‐8 without any presence of other components, which indicates the complete W‐POM entrapment within the crystals of the ZIF‐8. Upon calcination, the typical signals of ZIF‐8 disappeared, and two peaks representing the (111) and (200) facets from tungsten nitrides are clearly observed, confirming the successful conversion of all samples from W‐POM@ZIF‐8 precursors to W SAs/WNNC (Figure 2a). In addition to the XRD phase analysis results, the presence of W‐POM inside the ZIF‐8 crystals was confirmed by measuring the IR absorption peaks. The Fourier‐transform infrared (FTIR) spectrum obtained from the W‐POM@ZIF‐8‐5 is in good agreement with those of ZIF‐8, but no characteristic peak of W‐POM at around 1100 cm^−1^ is detected, which is taken for the coverage of ZIF‐8 (Figure S2, Supporting Information).

We reveal using scanning electron microscopy (SEM) the size and morphology of the as‐synthesized materials (Figures S3 and S4, Supporting Information). Although the amounts of the entrapped W‐POM vary, the as‐prepared precursors and ZIF‐8 have similar polyhedral structures with a dimension of ≈100 nm. Taking W SAs/WNNC‐5 as an example, the surface of the synthesized polyhedral products become rougher after conversion (Figure 2b), and a porous nanostructure of W SAs/WNNC‐5 is verified using transmission electron microscopy (TEM) and high‐angle annular dark‐field scanning transmission electron microscopy (HAADF‐STEM; Figure S5, Supporting Information; Figure 2c). The corresponding STEM energy‐dispersive X‐ray spectroscopy (EDS) mapping image that indicates a uniform elemental distribution of W and N (Figure 2d). In fact, the same morphology transformation has been observed in the catalysts with higher W‐POM loadings, (Figure S6a–c, Supporting Information). However, as the W‐POM loading increases, so does the concentration of overlapping nanoparticles. For W SAs/WNNC‐5, the ensembled W single atoms were marked with red circles, which are clearly found around the neighboring W_3_N_4_ nanoparticles (Figure 2e). Whereas less W single atoms were discovered in W SAs/WNNC‐10 (Figure S6d, Supporting Information), and almost no single atoms in WNNC‐20 and WNNC‐40. As shown by the Brunauer–Emmett–Teller (BET) analysis, catalysts with single atoms have larger specific surface area (Table S1, Supporting Information). The atomic resolution STEM‐HAADF image shows that the lattice distance is 0.24 nm, which is consistent with the (111) plane of W_3_N_4_ (Figure 2f).

We conducted X‐ray photoelectron spectroscopy (XPS) to study the valence states and the electronic interaction of the surface elements. The full scan spectrum of W SAs/WNNC‐5 confirms the presence of tungsten, nitrogen, and carbon (Figure S7a, Supporting Information). Regarding the W 4f region, two pronounced peaks, at the respective binding energy of 31.93 and 34.21 eV, could be assigned to tungsten nitrides, corresponding to the core levels of W 4f_7/2_ and W 4f_5/2_ (Figure S7b, Supporting Information). From the high‐resolution XPS spectra of N 1s, one peak at 398.7 eV is identified to represent N‐W bonding, and the fitted two peaks at 401.3 and 404.5 eV are allocated to the N‐C bonding (Figure S7c, Supporting Information). This further reinforces the presence of chemical bonding between W species and the carbon substrate. The W L3‐edge X‐ray absorption near edge structure (XANES) spectra of all the samples are given in Figure S8a,b (Supporting Information). The higher white line (≈10 209 eV) intensity observed from W SAs/WNNC‐5 and W SAs/WNNC‐10 suggests the W inside the single atom and nanoparticle ensembles has higher average oxidation states compared with those of WNNC‐20 and WNNC‐40.^[^ ^26^, ^27^ ^]^ From the Fourier transformation (FT) extended X‐ray absorption fine structure (EXAFS) (Figure S8c, Supporting Information), we attribute the peak located at 2.6 Å to the W–W coordination in tungsten nitrides, whereas the peak at around 1.95Å suggests the W‐N/C coordination that can be observed in both tungsten single atoms and tungsten nitrides.^[^ ^26^, ^27^ ^]^ The X‐ray absorption results are in agreement with the previous conclusion obtained from XPS and STEM measurements; further proving the successful synthesis of the binary system.^[^ ^24^ ^]^

### 2.3 Electrochemical Measurements

**Figure 3:**
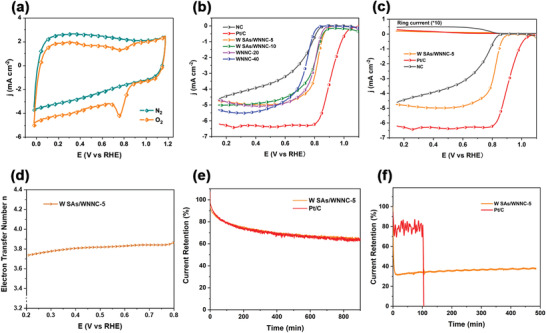
Electrochemical characterization electrocatalysts. a) CV curves of W SAs/WNNC‐5 in O_2_‐saturated and N_2_‐saturated 0.1 m KOH. b) Comparison of the oxygen reduction polarization curves at a rotating speed of 1600 rpm. c) RRDE voltammograms were recorded for different catalysts in O_2_‐saturated 0.1 m KOH at 1600 rpm. d) Electron transfer number at the W SAs/WNNC‐5 electrode based on the RRDE result. e) Comparison of current retention‐time response curves in O_2_‐saturated 0.1 m KOH solution for 16 h. f) Comparison of current retention‐time response curves in MeOH solution for 13 h.

The cyclic voltammetry (CV) and linear sweep voltammetry (LSV) curves were collected to analyze the ORR catalytic activities. A three‐electrode workstation with O_2_‐saturated 0.1 m potassium hydroxide (KOH) was employed. All potentials were calibrated to the reversible hydrogen electrode (RHE). Pt/C and NC (ZIF‐8 derived catalyst) were also evaluated as the benchmark and control samples. The CV curves were obtained after activation, where the W SAs/WNNC‐5 affords a more positive reduction peak of 0.78 V, which is comparable to Pt/C catalyst, and higher than that of NC catalyst (**Figure** **3**a; Figure S9, Supporting Information). Using rotating ring disk electrode (RRDE) measurements, we reveal that W SAs/WNNC‐5 has the closest onset potential to Pt/C (0.89 V vs 1.03 V), and its half‐wave potential can reach 0.83 V (Figure 3b). This finding demonstrates a relatively high catalytic activity among the series of W‐based catalysts and other ORR catalysts. Refs. [31, 32, 33, 34, 35, 36] (Table S2, Supporting Information).

We show the oxygen reduction polarization curves of other catalysts collected with different rotating speeds (Figure S10, Supporting Information). Notably, as the W‐POM loading increases, the onset potentials of the derived W SAs/WNNC catalysts demonstrate a negative trend. We suspect that this is a result of the poor dispersion of W_3_N_4_ nanoparticles and the lack of W single atoms with increased W‐POM loading, as discussed earlier. Likewise, in comparison with Pt/C and NC catalysts, W SAs/WNNC‐5 gives the smallest ring current density (≈0.023 mA cm^−2^) for H_2_O_2_ oxidation (Figure 3c), indicating a promising ORR selectivity. A selective four‐electron pathway per oxygen molecule is determined from the electron transfer number close to 4 on W SAs/WNNC‐5 electrode, thus, a highly effective ORR process was enabled (Figure 3d). The stability is equally important for catalytic performance, and the W SAs/WNNC‐5 catalyst exhibits a similar current retention ratio with that of Pt/C during the long‐term chronoamperometric tests, as well as a much better methanol crossover tolerance (Figure 3e,f).

**Figure 4:**
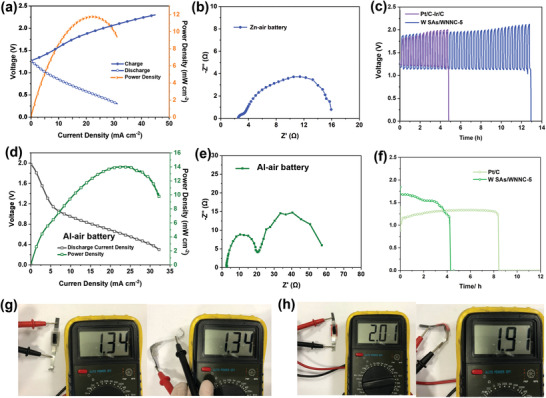
Electrochemical performance of the all‐solid‐state a–c) Zn–air batteries and d–f) Al‐air batteries. a) Discharge–charge polarization curve and power density curves of the solid‐state Zn–air batteries. b) Impedance curve of the solid‐state Zn−air batteries testing at 1.5 V. c) Cycling stability of the Zn–air batteries using W SAs/WNNC‐5 and Pt/C‐Ir/C as the air cathodes. d) Current density and power density of the solid‐state Al−air batteries. e) Impedance curves of the solid‐state Zn−air batteries at 1.5 V. f) Discharging stability of the Al–air batteries using W SAs/WNNC‐5 and Pt/C as the air cathodes. Digital images of the assembled solid‐state g) Zn–air batteries and h) Al–air batteries with the same voltage measured using a voltammeter when at flat state and bending state.

Given the attractive catalytic activity and ORR selectivity, W SAs/WNNC‐5 and the mixture of Pt/C and Ir/C (1:1) were evaluated as the electrode materials for their performance in all‐solid‐state Zn–air batteries, and W SAs/WNNC‐5 and Pt/C for Al–air batteries. The charge–discharge polarization curves of Zn–air battery are shown in **Figure** **4**a. The solid‐state Zn–air cell with W SAs/WNNC‐5 cathode delivers the largest power density of the 11.7 mW cm^−2^ and a relatively small resistance (Figure 4b). A stable 12 h cycling plateau with the voltage window between 1.2 and 2 V is shown in Figure 4c, revealing the superior stability of the electrode material, which is longer than the battery with Pt/C–Ir/C electrode (Table S3, Supporting Information). Refs. [37, 38, 39, 40, 41, 42] For solid‐state Al–air battery application, we demonstrate a similar power density and current density as the Zn–air battery (Figure 4d). This finding indicates a lower resistance in Al–air battery using W SAs/WNNC‐5 as the air cathode material (Figure 4e). In addition, we also show a high open circuit‐voltage and long discharge up to 250 min, competitive in comparison with other reported work (Figure 4f; Table S4, Supporting Information). Refs. [40, 43, 44, 45, 46, 47]. A higher discharge voltage was also observed compared to those Al‐air batteries with the Pt/C air cathodes. Notably, both metal‐air batteries can generate almost the same open‐circuit voltage (OCV) under flat and bent conditions, indicating good resistance toward mechanical deformation (Figure 4g,h).

### 2.4 DFT Calculations

**Figure 5:**
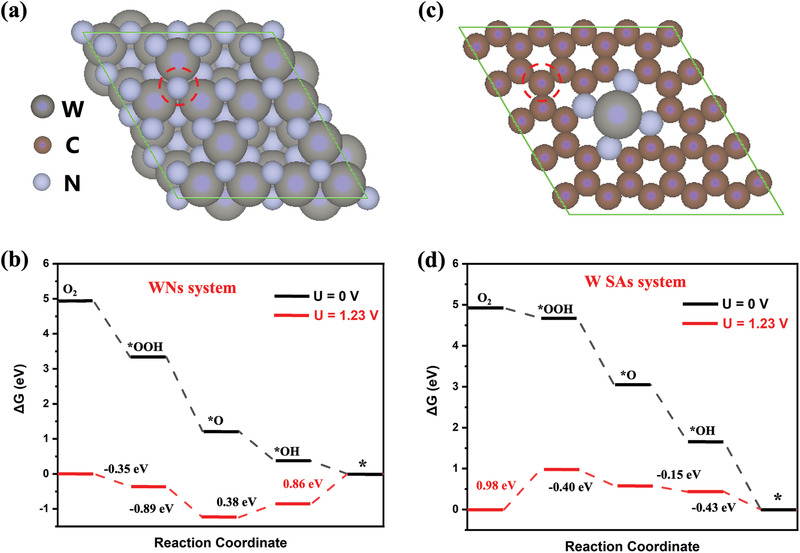
a) Structural model of N‐terminal W_3_N_4_ (111) facet, and b) The Gibbs free energy diagram of ORR in the WNs system c)Structural model of WN_4_‐G, and d) The Gibbs free energy diagram in the W SAs system.

In order to understand the underlying mechanism of the improved ORR performance of the ensemble W SAs/WNNC‐5, we performed density‐functional theory (DFT) calculations to simulate both W_3_N_4_ nanoparticles (WNs) and W single atoms (W SAs) electrocatalytic performance. For the WNs, the (111) facet (Figure 5a) was considered because the XRD and STEM results indicate that it is the most exposed surface. As for W SAs, the WN_4_ coordination (Figure 5c) was here considered since it has been reported thermodynamically favorable.^[^ ^28^ ^]^ The Gibbs free energy evolution along the 4‐electron ORR pathway was determined under the computational hydrogen electrode model.^[^ ^29^ ^]^

For the WNs, we show that the three‐fold N atom is the most favorable adsorption site (Figure 5a; red circle). As can be seen from the Gibbs free energy diagram of ORR on the W_3_N_4_ (111) facet (Figure 5b), the surface N sites can readily activate O_2_ and thus facilitate the subsequent hydrogenation step. However, the last two steps (*O→*OH and *OH→H_2_O) are uphill in energy, with the latter step being the potential‐determining step (PDS). We summarize a more detailed discussion including other potential structural models considered in this study in Figures S11–S13 (Supporting Information).

With regard to W SAs, our calculations indicate that the most likely active adsorption site is the neighboring C atom instead of the central W atom (Figures S14–S20, Supporting Information). In this case, the O_2_ activation is found to be the PDS, whereas all the subsequent steps are downhill in energy at *U* = 1.23 V (Figure 5d). Thus, the W SAs and WNs have different PDSs in ORR and could allow for synergy when active sites are intimately blended.

We show that the widely observed scaling relation between *OOH and *OH also holds for both the single WNs and W SAs system (i.e., *G*(*OOH) ≈ *G*(*OH) + 3 eV at *U*  =  0 V) (Figure 5c,d).^[^ ^30^ ^]^ Nevertheless, our calculations reveal that they exhibit different capabilities of stabilizing ORR intermediate adsorbates and thus have different potential‐determining steps, as elaborated in the above discussion. Therefore, when WNs and W SAs are purposefully integrated, one may expect that they could complement each other to synergistically catalyse ORR along the reaction pathway, thereby leading to an improved catalytic performance observed in the ensemble W SAs/WNNC‐5.

## 3 Conclusion

In summary, we demonstrate the synergistic combination of the W single atom and W_3_N_4_ nanoparticle (W SAs/WNNC) as efficient catalysts for ORR, successfully prepared by a new oversaturation method to complement the complete cage‐confinement route. With the high catalytic activity for ORR by the synergistic combination, we also demonstrate a remarkable performance as metal‐air battery cathodes. Our DFT calculations suggest that the combination of single atoms and nanoparticles provides a significant opportunity to improve the catalytic performance through bypassing the scaling relations using simple catalytic materials. The present work expands the library of synthesized combinations of single atoms and nanoparticles, which represents a novel material design for wider catalytic applications.

## Conflict of Interest

The authors declare no conflict of interest.

## Supporting information

Supporting Information

## Data Availability

Research data are not shared.
