# Supplementary material for: Localizing Tungsten Single Atoms around Tungsten Nitride Nanoparticles for Efficient Oxygen Reduction Electrocatalysis in Metal–Air Batteries
Source: Adv Sci (Weinh). 2022 Jun 22;9(28):2105192. doi: 10.1002/advs.202105192 (PMC9534944; doi:10.1002/advs.202105192)
Supplement: Supplementary file 1 — Supporting Information [file ADVS-9-2105192-s001.pdf]

WILEY-VCH

ADVANCED SCIENCE
Open Access

Supporting Information

for *Adv. Sci.*, DOI: 10.1002/advs.202105192

Localizing Tungsten Single Atoms Around Tungsten Nitride Nanoparticles for Efficient Oxygen Reduction Electrocatalysis in Metal-Air Batteries

Yuanyuan Ma, Yong Yu, Junhui Wang, Jason Lipton, Hui Ning Tan, Lirong Zheng, Tong Yang, Zhaolin Liu, Xian Jun Loh, Stephen J. Pennycook, Lei Shen*, Zongkui Kou*, André D. Taylor*, John Wang**

Supporting Information

Localizing Tungsten Single Atoms Around Tungsten Nitride Nanoparticles for Efficient Oxygen Reduction Electrocatalysis in Metal-Air Batteries

Yuanyuan Ma, Yong Yu, Junhui Wang, Jason Lipton, Hui Ning Tan, Lirong Zheng, Tong Yang, Zhaolin Liu, Xian Jun Loh, Stephen J. Pennycook, Lei Shen*, Zongkui Kou*, André D. Taylor*, John Wang**

Dr. Y. Y. Ma, Y. Yu, J. H. Wang, H. N. Tan, Prof. S. J. Pennycook, Prof. J. Wang

Department of Materials Science and Engineering

Faculty of Engineering

National University of Singapore, 117574, Singapore

E-mail: msewangj@nus.edu.sg;

Dr. Y. Y. Ma, J. Lipton, Prof. A. D. Taylor

Department of Chemical and Biomolecular Engineering

Tandon School of Engineering

New York University, Brooklyn, NY 11201, USA

Email: andre.taylor@nyu.edu

J. Lipton

Chemical Sciences and Engineering Division

Argonne National Laboratory

Lemont, IL 60439, USA

Dr. L.R. Zheng

Beijing Synchrotron Radiation Facility Institute of High Energy Physics, Chinese Academy of Sciences, Beijing 100049, China

Email: zhenglr@ihep.ac.cn

Dr. T. Yang

Department of Applied Physics

The Hong Kong Polytechnic University, Hung Hom, Hong Kong, P. R. China

Dr. Z. L. Liu, Prof. X.J. Loh

Institute of Materials Research and Engineering
Agency for Science Technology and Research (A* STAR), 2 Fusionopolis Way, Innovis 138634, Singapore

Prof. L. Shen

Department of Mechanical Engineering

National University of Singapore, 117575, Singapore

E-mail: shenlei@nus.edu.sg

Prof. Z. K. Kou,

State Key Laboratory of Advanced Technology for Materials Synthesis and Processing, Wuhan University of Technology, Wuhan 430070, P. R. China

E-mail: zongkuikou@whut.edu.cn

1. Experimental Details

1.1 Chemicals

Zinc nitrate hexahydrate ($Zn(NO_3)_2$•$6H_2O$), 2-methylimidazole (2-MIM), methanol, and Nafion (5 wt %) solution were purchased from Sigma-Aldrich. Tungsten polyoxometalate (W-POM) was purchased from Fluka. They were all used as the received without further purification. Deionized (DI) water was obtained via on-site purification in our laboratory.

1.2 Preparation of the W-POM@ZIF-8-x precursors and WNNC-x products.

The W-POW@ZIF-8 was synthesized by the coordination reaction of Zn ions and 2-MIM. In a typical synthesis, 0.369 g of $Zn(NO_3)_2 \cdot 6H_2O$ and 0.814 g of 2-MIM were dissolved into 12.5 mL of methanol separately under magnetic stirring to form clear solutions, denoted as solution A and solution B. At the same time, varied amount of W-POM was added into 5 mL of DI water to form a homogeneous solution C. Next, the solutions A and C were poured into solution B together, and the mixture was then stirred vigorously for 2.5 hours to *in-situ* grow the W-POM@ZIF-8. The W-POM@ZIF-8 was then separated from the solution by centrifugation and thoroughly washed once with DI water and twice with methanol before finally dried overnight at 80 °C in the vacuum oven. A series of W-POM@ZIF-8 with different amounts of W-POM added was prepared, denoted as W-POM@ZIF-8-x (where x is the amount of W-POM, in mg, in the following values: 5, 10, 20, 40).

After drying, the products were annealed in a furnace at 900 °C with a ramping rate of 5 °C min^{-1} under continuous N_2 flow for 3 hours. As a result, the pyrolysis process rendered the conversion of W-POM@ZIF-8-x into W SAs/WNNC-x and WNNC-x. The ZIF-8 and pyrolyzed products (NC) without W_3N_4 were synthesized as the control examples.

1.3 Materials Characterizations

The size and morphology of samples were performed using Scanning electron microscopy (SEM) (Zeiss Supra 40 FE SEM) with samples placed on aluminum foil held by copper tape to the sample holder. High angle aberration-corrected dark-field scanning transmission electron microscopy (HAADF-STEM) was carried out with a JEM 1011 transmission electron microscope operated at 200 kV. Powder X-ray diffraction was conducted on the Bragg-Brentato Theta-theta Diffractometer (Cu Kα radiation, λ = 0.15418nm) to study the crystal structures of samples. X-ray photoelectron spectroscopy (XPS) was carried out for chemical composition information. Fourier-transform infrared spectroscopy (FTIR) was conducted on the Agilent CARY 660 FTIR Spectrometer with samples prepared as thin KBr pellets at wave numbers ranging from 4000 to 400cm^{-1}, tested against the empty

sample holder accounting for background wavelength. The X-ray absorption fine structure spectra data were recorded at the 1W1B station of the Beijing Synchrotron Radiation Facility. All measurements were taken at room temperature with the operating voltage at 2.5 GeV and a maximum current of 250 mA. The samples were pelletized into disks with a diameter of 13 mm and a thickness of 1 mm using BN powder as the binder. Athena software was used to process and analyze the acquired data. The mass of electrode materials was recorded by an AX/MX/UMX Balance (METTLER TOLEDO, maximum = 5.1 g; delta = 0.001 mg).

1.4 Electrochemical measurements

Electrochemical measurements were investigated by a three-electrode rotator electrochemical workstation (Wave Vortex) equipped with a rotating ring disk electrode (RRDE) apparatus. The electrochemical oxygen reduction reaction (ORR) activities were performed in 0.1M potassium hydroxide (KOH) solution (pH≈12.56) purged with O_2 or N_2 at room temperature. Graphite rod and leak-free Ag/AgCl electrode were employed as the counter and reference electrodes, respectively. All potentials subsequently used in this work were referenced to the reversible hydrogen electrode (RHE), based on the equation (1):

$$E_{RHE} = E_{Ag/AgCl} + 0.059 * pH + 0.199 \quad (1)$$

The ink for working electrodes were prepared as follows: 10 mg of sample was firstly dispersed into 500 µl of DI water, and then 480 µl of a solution (69.56% water, 27.82% ethanol, 2.615% of Nafion) and 20 µl of Nafion were added under sonication to form a homogeneous ink. The above ink was then dripped on a glassy carbon RDE to achieve a mass loading of 1.62 mg cm^{-2}. 20 wt% Pt/C has a lower catalyst loading of 0.4 mg cm^{-2} was also prepared for benchmark. The electrochemical activity of catalysts was studied using linear sweep voltammetry (LSV) at a scan rate of 5 mV/s and various rotation speeds. Cyclic voltammetry was carried out to activate the electrocatalysts before measuring the performance at a rate of 50 mV/s. Nyquist plots were obtained on the same one glassy carbon electrode and in the O_2-saturated 0.1 M KOH electrolyte from 1 MHZ to 0.1 HZ. All polarization

curves were corrected for background current tested in N_2-saturated environment. For the RRDE measurements, catalyst inks and electrodes were prepared by the same method as for RDE, and the ring potential was constant at 1.3 V vs RHE.

1.5 Calculation of electron transfer number (n) and and %HO_2^- for oxygen reduction reaction

On the basis of RRDE results, the ratio of HO_2^- formation (p) is defined as the fraction of O_2 reduced to HO_2^- and can be calculated by the equation (2):

$$p = 2 * \frac{\frac{i_r}{N_C}}{i_d + \frac{i_r}{N_C}} \qquad (2)$$

The equation used to calculate the electron transfer number (n) is equation (3):

$$n = 4 * \frac{i_d}{i_d + \frac{i_r}{N_C}} \qquad (3)$$

where n is the electron number, i_d is the disk current, i_r is the ring current, and N_C is the collection efficiency of the RRDE (here is 0.37), which is defined as the fraction of product from the disk to the ring.

1.6 Metal-air battery measurements

Zinc-air battery testing was carried out on an all-solid-state setup. The catalyst coated cathode for the all-solid-state setup was made as follows: 9 mg of sample mixed with 1.875 mg of carbon black and 20.23 µl of Nafion, and all of them then dispersed in the 4 ml ethanol by ultrasonic treatment to obtain the homogeneous solution. A piece of carbon cloth was used as the current collector (size: 1.5 × 0.5 cm^2) to impart mechanical flexibility, and the mass loading of catalyst was 3 ± 0.1 mg cm^{-2}. In a detailed fabrication process, air cathode, Ti mesh, solid-state electrolyte, and Zn foil, in order, were bonded between two pieces of flexible acrylic covers (3M acrylic tape, 1 mm thick). An air inlet (1.5 ×

0.4 cm^2) was made in the center of the acrylic at the air cathode side whilst another inserted piece of acrylic tape was cut to form a well (1.5 × 0.5 cm^2) to contain the solid electrolyte. The solid electrolyte was synthesized by the polymerization process. First, 10 ml of electrolyte consisting of KOH (11.25 mol) and ZnO (0.25 mol) was mixed with 1 g of acrylic acid and 0.15 g N, N′-methylene-bisacrylamide. The white precipitate then was filtered out after 10-min stirring. Next, 100 µl of 0.3 M $K_2S_2O_8$ was dropped into the electrolyte solution, where 350 µl of the electrolyte solution was then poured straight away into the acrylic well once the solution started to polymerize.

Al-air battery performance was conducted by the same all-solid-state Zn-air battery setup, consisting of the Al foil anode (Alfa Aesar, size: 1.5 × 0.5 cm^2, thickness 0.1 mm), catalysts-coated carbon cloth as the air cathode, and solid-state electrolyte. However, the electrolyte was instead prepared from KOH (11 M), ZnO (0.0075 mol), Na_2SnO_3 (0.01 mol) and $In(OH)_3$ (0.0005 mol), which was mixed with 1 g of acrylic acid and 0.15 g N, N′-methylene-bisacrylamide. Before applying, the Al foil was immersed in 100 g L^{-1} NaOH solution to clear the oxidation layer on the surface.

Cycling tests for solid-state batteries were recorded at the constant current of 0.75 mA under room temperature around 25°C.

2. DFT Details

2.1 The details of DFT computational method

All calculations in our work were performed based on density functional theory using the Vienna Ab initio Simulation Package (VASP).[1] The projector augmented-wave (PAW) method was employed to describe the ion–electron interaction.[2,3] The electronic exchange and correlation interactions were approximated by the Perdew-Burke- Ernzerhof (PBE) functional.[4] The kinetic cutoff energy of the plane wave basis was set to 500 eV. Empirical atom-pairwise corrections proposed by DFT-D3 scheme with Becke-Jonson damping were applied to describe the long-range van der Waals interactions.[5,6]

A 2×2 supercell with 7 layers of W and N atoms (a = b = 11.86 Å) (Figure S9 and S10) was used as the structural model of WNs system. The top four layers were relaxed, while the bottom three layers were fixed in the structural relaxation. For the W SAs system, we use a 5×5 graphene supercell (a = 12.46 Å and b = 12.27 Å), in which six C atoms are replaced by four N atoms and one W atom, forming a WN_4-G model (Figure S12). The Brillouin zone was sampled using the Monkhorst-Pack scheme[7] with a k-mesh of 6×6×1 for both W single atom and W_3N_4 NPs system. A vacuum of 20 Å along the z-direction was used to prevent spurious interaction between the periodically repeated images. For geometry optimization, all atoms were fully relaxed until the residual forces on each atom converged to 0.01 eV $Å^{-1}$. The energy convergence criterion was set to 1×10^{-5} eV.

The free-energy change (ΔG) of each ORR elemental step was calculated by

$$\Delta G = \Delta E + \Delta ZPE - T\Delta S + \Delta G_U$$

where ΔE is the reaction energy calculated based on DFT calculations, ΔZPE is the change of the zero-point energy, T is the temperature (298.15 K), and ΔS is the change of entropy. ΔG_U and ΔG_{PH} are the contributions to the free energy due to the change of the electrode potential (U) and the pH value, respectively. $\Delta G_U = -neU$, where n is the number of transferred electrons and U is the potential at the electrode. From the thermodynamic aspect, the positive ΔG implies the presence of the barrier and an extra voltage is needed to drive the process, while a negative value indicates a spontaneous reaction step.[8]

The ORR overpotential (η^{ORR}) was calculated by

$$\eta^{ORR} = 1.23\ V + G_{max}/e$$

where 1.23 V is the equilibrium potential of the overall four-electron ORR for pH = 0 at T = 298.15 K, and ΔG_{max} is the largest free energy change among the four reaction steps.

2.2 Identify the correct active sites.

In our work, W_3N_4 nanoparticles (WNs) system was firstly studied. According to the experimental XRD and STEM results, the (111) facet was chosen to calculate the Gibbs free energy diagram of W_3N_4 nanoparticles. The facet could be either W- (Figure S9) or N- (Figure S10) terminated, which are hereafter denoted as the W atom layer and the N atom layer, respectively.

In view of this, initially W atom was investigated for the (111) facet. After investigating all possible adsorption configurations and reaction pathways for W atom in WNs system, we concluded that the W atom is unlikely to be an appropriate active site for ORR. In some structural models, as shown in Figure S11, the adsorption of ORR intermediates was not stable, or even induced surface breakdown. In the remaining structural models, the ORR overpotentials were so large as to rule out the possibility of ORR occurring. The poor catalytic performance for W atoms in the WNs system was caused by the strong electronegativity of W atom, and similar phenomenon also occurred in W SAs system. According to a previous report,[9] the W single atom coordinated with four N atoms and doped on the graphene substrate (WN_4-G) was considered in the W single atoms (W SAs) system (Figure S12 a and b). Given the large electronegativity, W atom exhibited strong adsorption for oxygen-containing intermediates, such as *O or *OH, promoting the dissociation step of *OOH into *O and *OH (Figure S12c-h). Therefore, dissociative ORR mechanism was the only possible and reasonable pathway for W SAs system as the *OOH adsorbate tends to dissociate spontaneously (Figure S13). However, the computational results (Figure S14) suggest that W atom was also not a good active site for ORR in W SAs system.

Having ruled out the possibility that W atom can drive a fast ORR, we proceeded with the N atom layer model of WNs. There are two types of surface N atoms, namely the N atom bonded with three W atoms (N1) and the N atom coordinated with two W atoms (N2), as shown in Figure S10. The Gibbs free energy diagram of ORR on N1 and N2 structures (Figure 5a and S15) illustrated that N1 exhibited

better catalytic performance than N2. Therefore, in the present work, N1 was selected as the active sites.

For W SAs system, the possible ORR active center was tested and confirmed by DFT calculation results. The stability of *OH adsorption on eight different atoms including seven C atoms and one N atom (Figure S16) were investigated under two different conditions: W atom passivated with or without OH^-. All simulation results illustrated that, if the active sites were close to W atom, the *OH adsorbed on these active sites, including N, C1, C2, C3 and C4 atoms, will move to the top site of W atom, no matter whether W was passivated or not (Figure S17). Among the stable adsorbed configuration for *OH adsorption, including C5, C6 and C7, the adsorption strength of *OH on C5 atom was the weakest (Table S5), resulting in a highest ORR activity (Figure S18). Considering the ORR in our experiment was conducted in alkaline solution, the W atom in the structural models which was investigated for ORR in W SAs system was passivated by OH^-, and then C5 atom acted as the active sites to conduct the ORR calculation.

3. Supplementary Figures and Tables

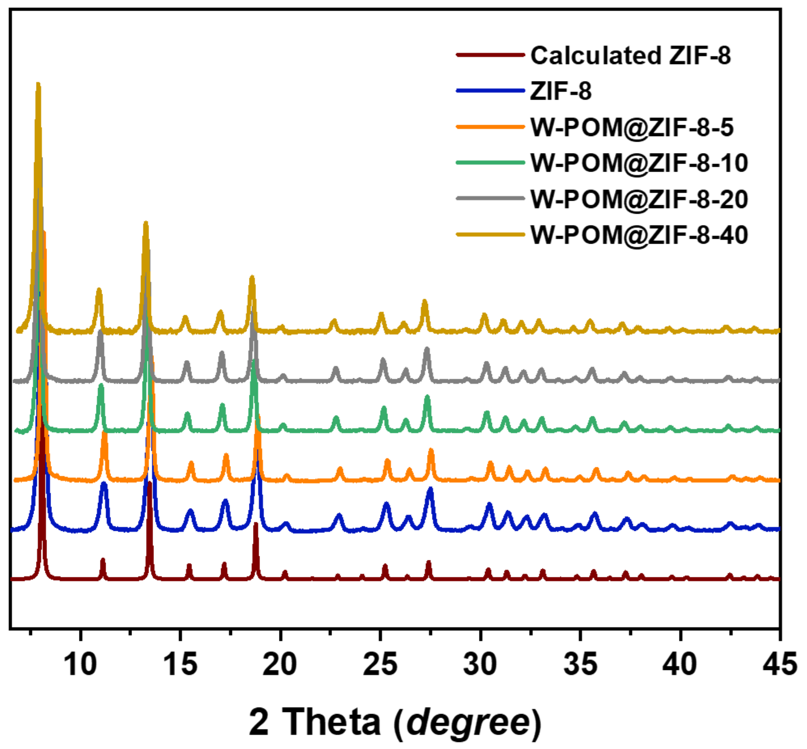

Figure S1. XRD curves of all precursor samples.

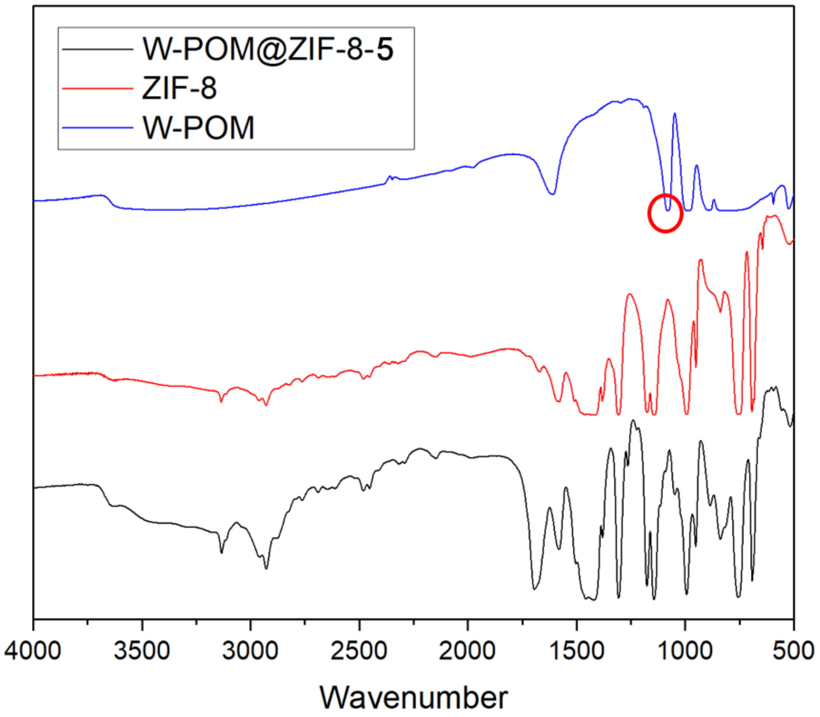

Figure S2. FTIR Spectra of W-POM@ZIF-8-5, ZIF-8, and W-POM respectively.

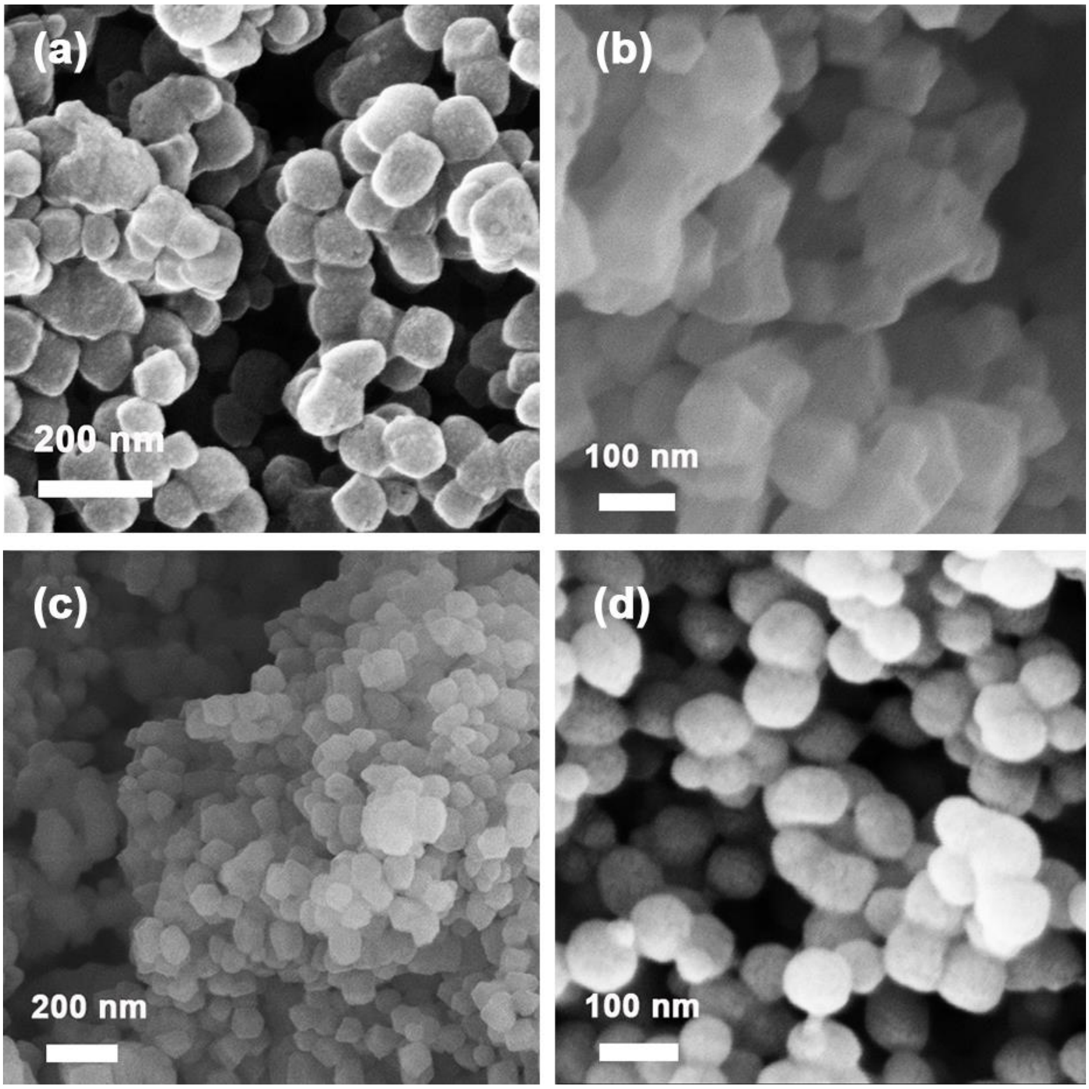

Figure S3. SEM image of (a)W-POM@ZIF-8-5, (b)W-POM@ZIF-8-10, (c)W-POM@ZIF-8-20, and (d)W-POM@ZIF-8-40.

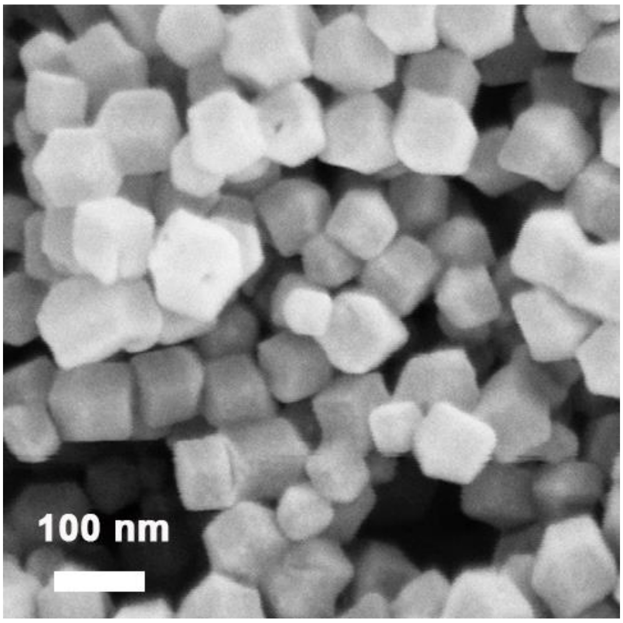

Figure S4. SEM image of ZIF-8

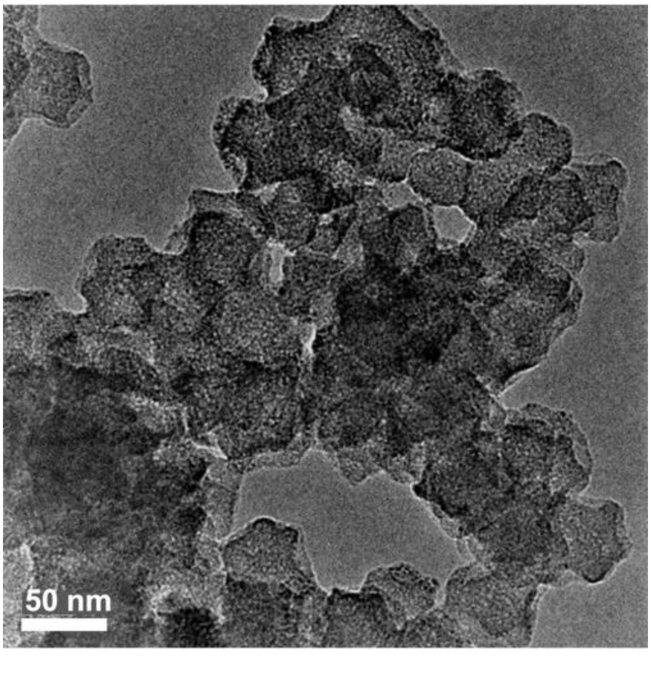

Figure S5. TEM image of W SAs/WNNC-5

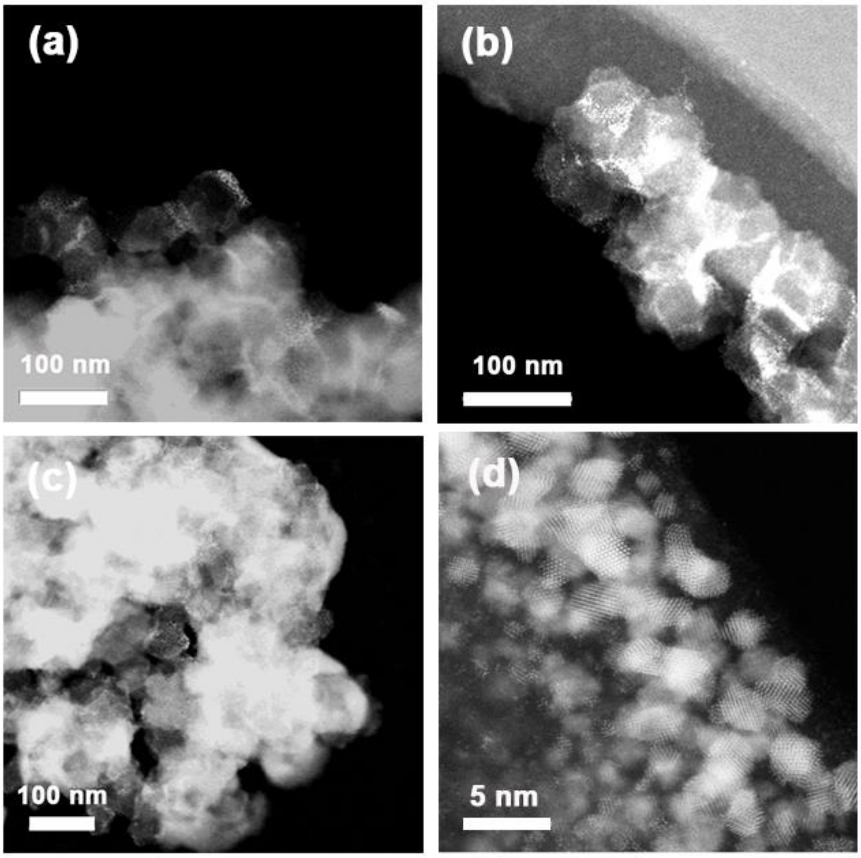

Figure S6. HAADF-STEM images of (a) W SAs/WNNC-10 (b) WNNC-20, and (c) WNNC-40. (d) HRSTEM images of W SAs/WNNC-10.

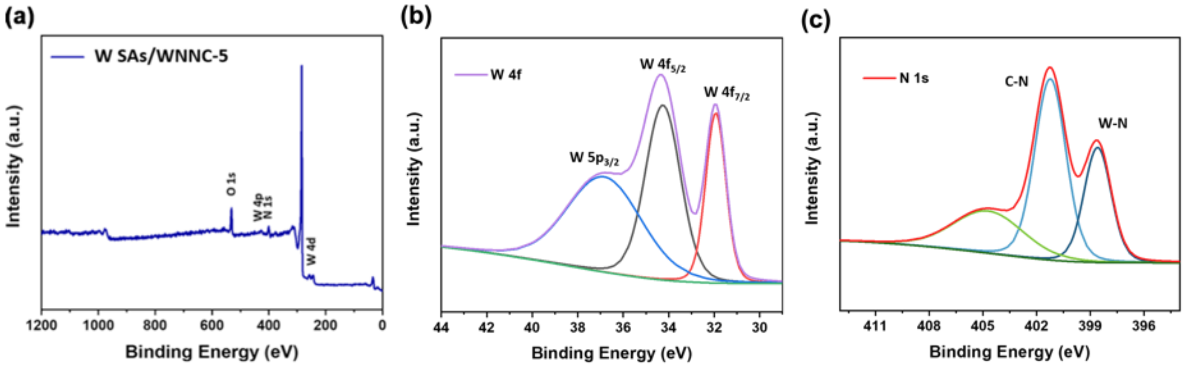

Figure S7. XPS spectra of W SAs/WNNC-5. (a) Full scan. (b) W 4f. (c) N 1s.

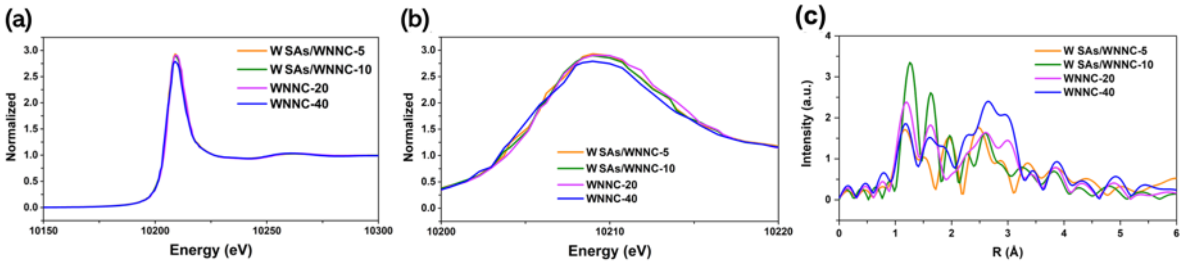

Figure S8. (a) The normalized W L3-edge XANES spectra (full range). (b) The normalized W L3-edge XANES spectra. (c) k^2 [χ(k)] weighted FT of EXAFS in R space.

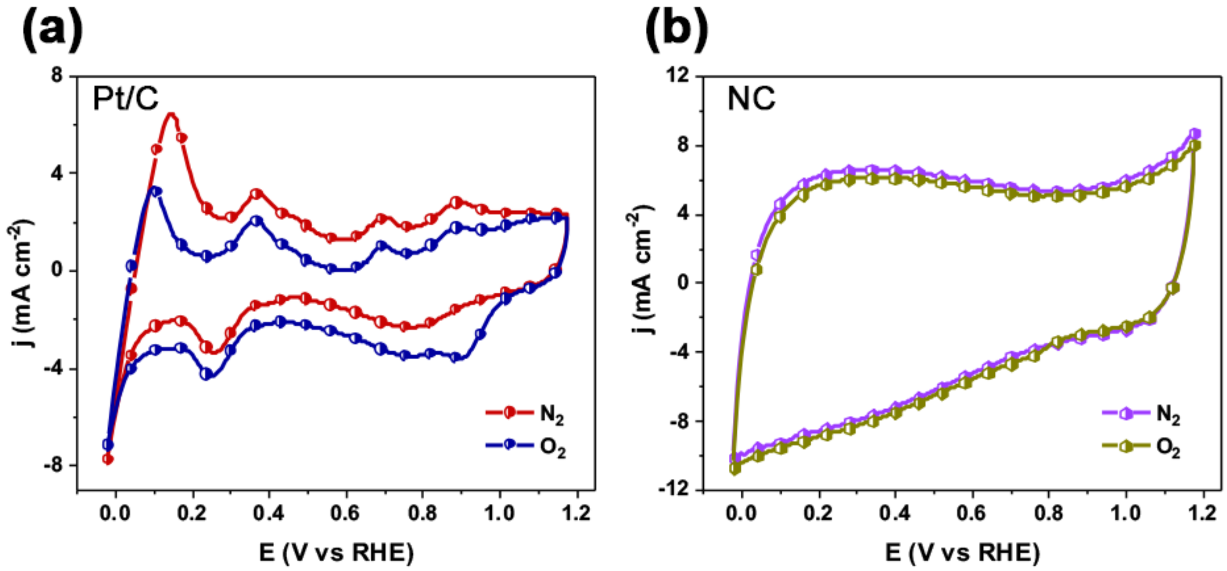

Figure S9. CV curves of (a) Pt/C and (b) NC in N_2-saturated and O_2-saturated 0.1 M KOH solutions.

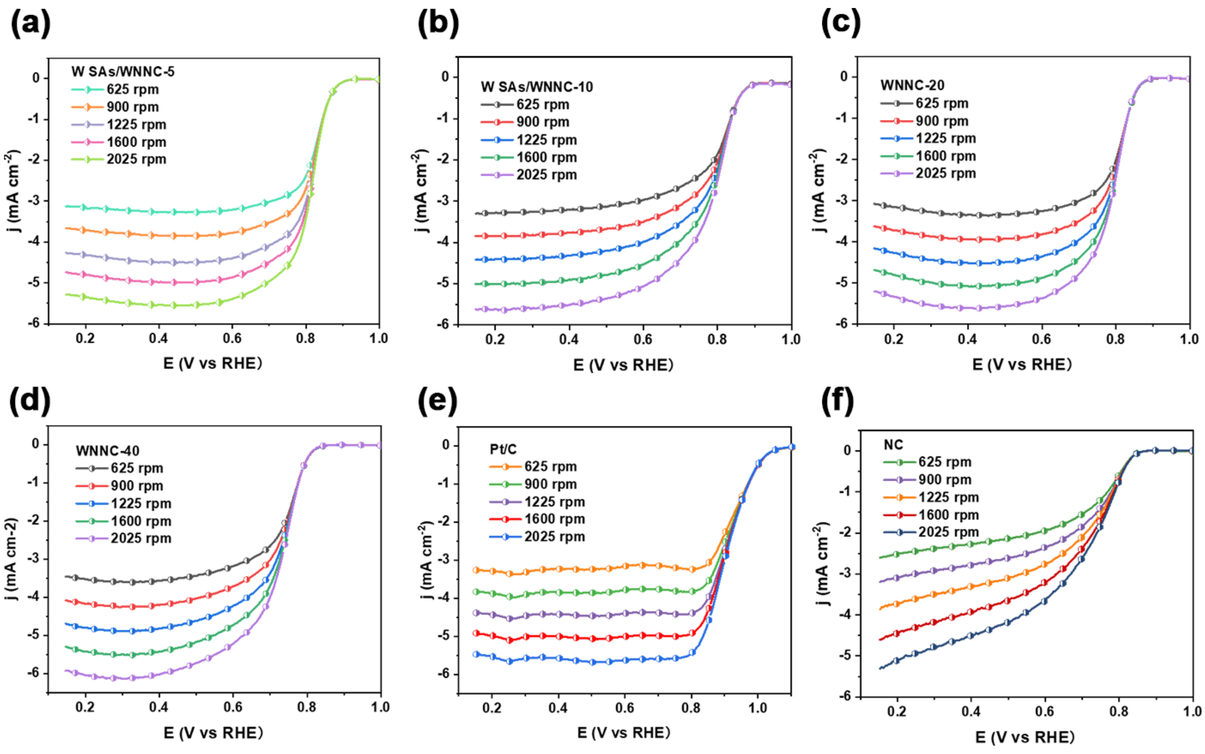

Figure S10. Oxygen reduction polarization curves of all catalysts at various rotating speeds.

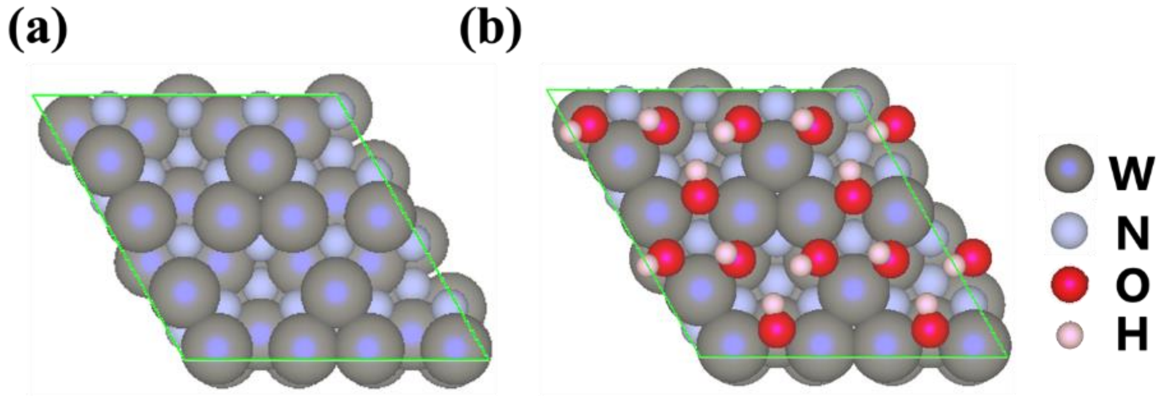

Figure S11. (a) The structural model of W atom layer in (111) facet. (b) The corresponding structural models for (a) with the passivation of all W atoms.

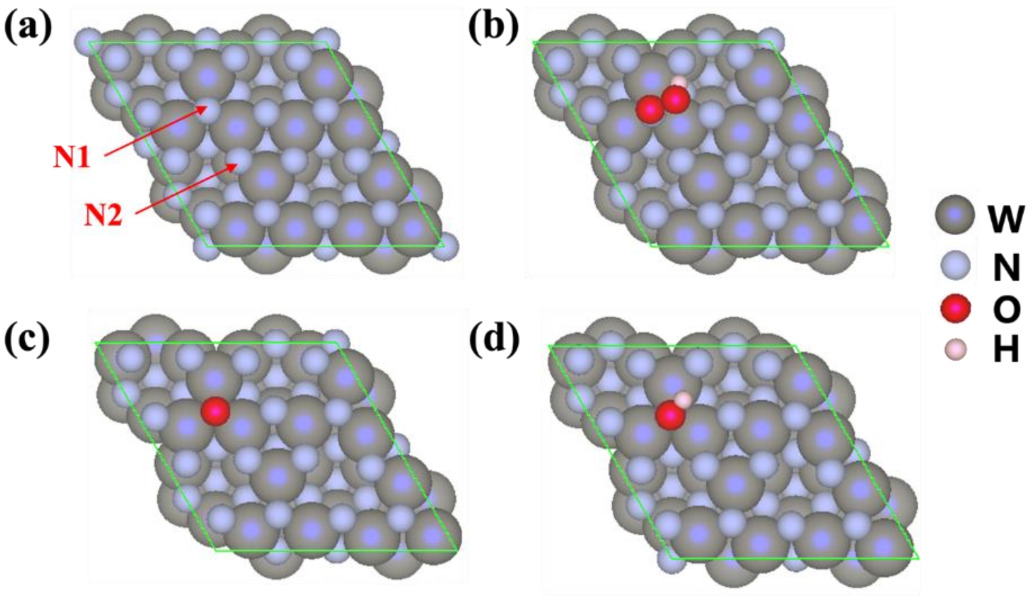

Figure S12. The structural models of: (a) N atom layer in (111) facet, (b) *OOH adsorption, (c) *O adsorption, and (d) *OH adsorption on N1 atom in the N atom layer in (111) facet.

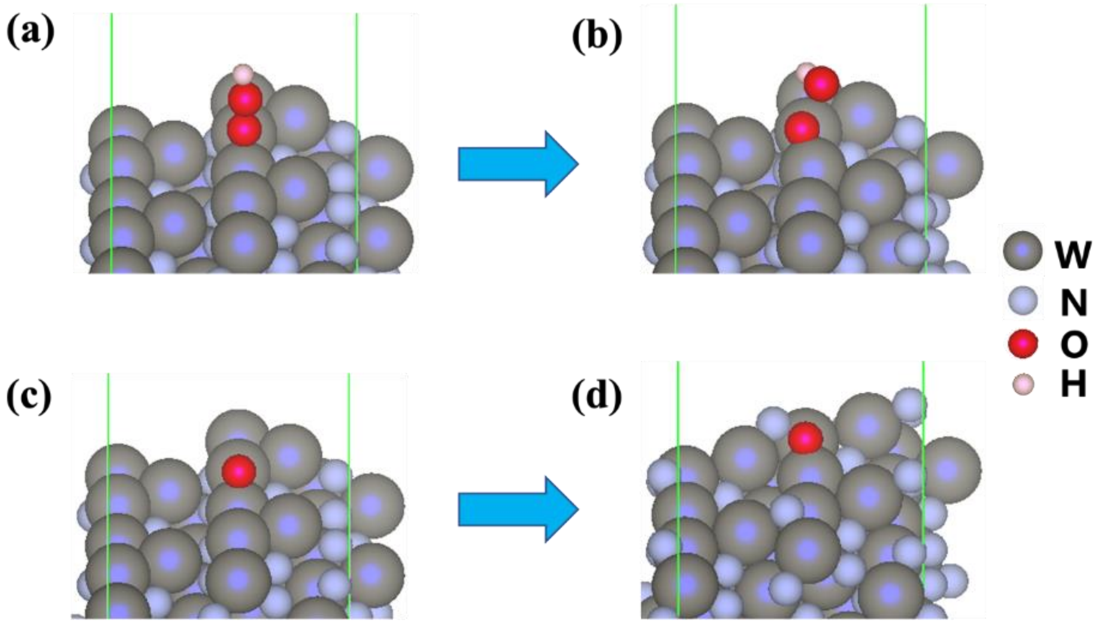

Figure S13. The structural models of (a), (b)*OOH adsorption and (c), (d) *O in W atom layer in (111) facet. (a), (c) before and (b), (d) after calculation.

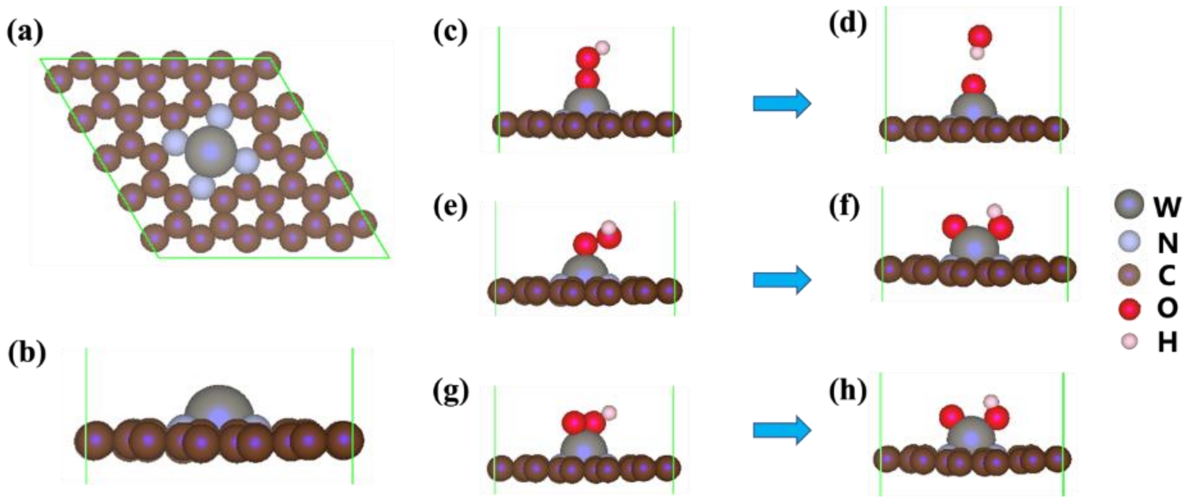

Figure S14. The (a) top view, and (b) side view for the structural model of W SAs system. The initial structural models for (c) end configuration 1, (e) end-configuration 2 and (g) side-configuration of *OOH adsorption on W SAs system before calculation. (d), (f) and (h) were the corresponding final structural models of (c), (e) and (g), respectively, after calculation.

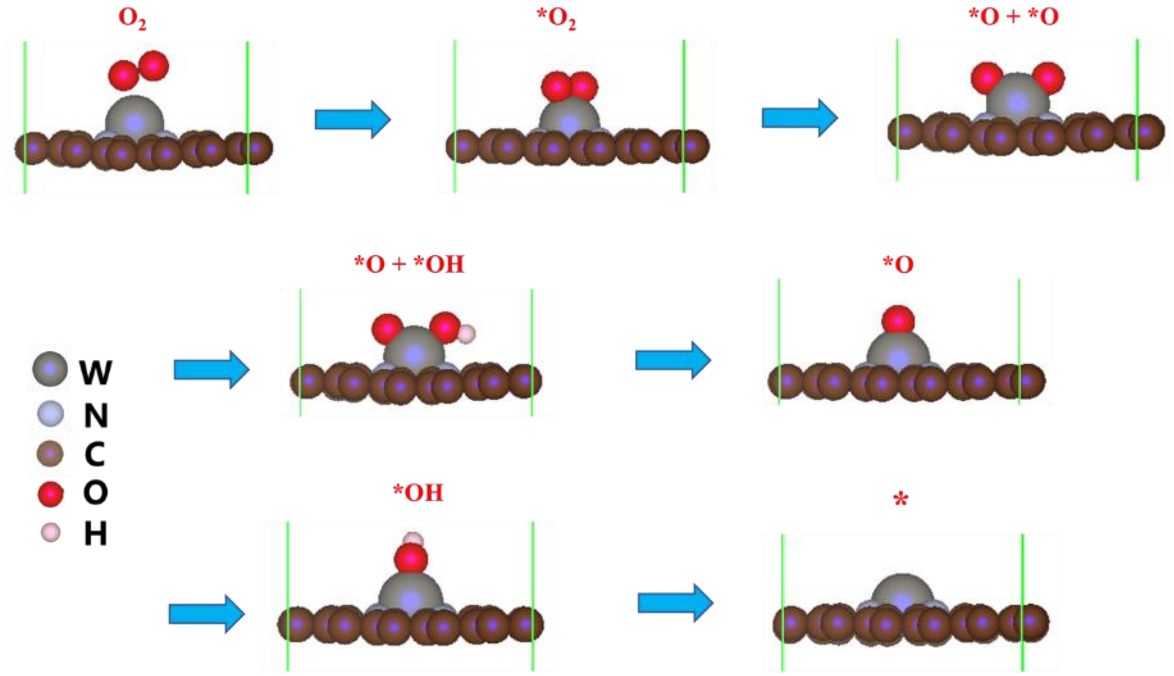

Figure S15. The structural model for each ORR step, when W atom as the active site in W SAs system.

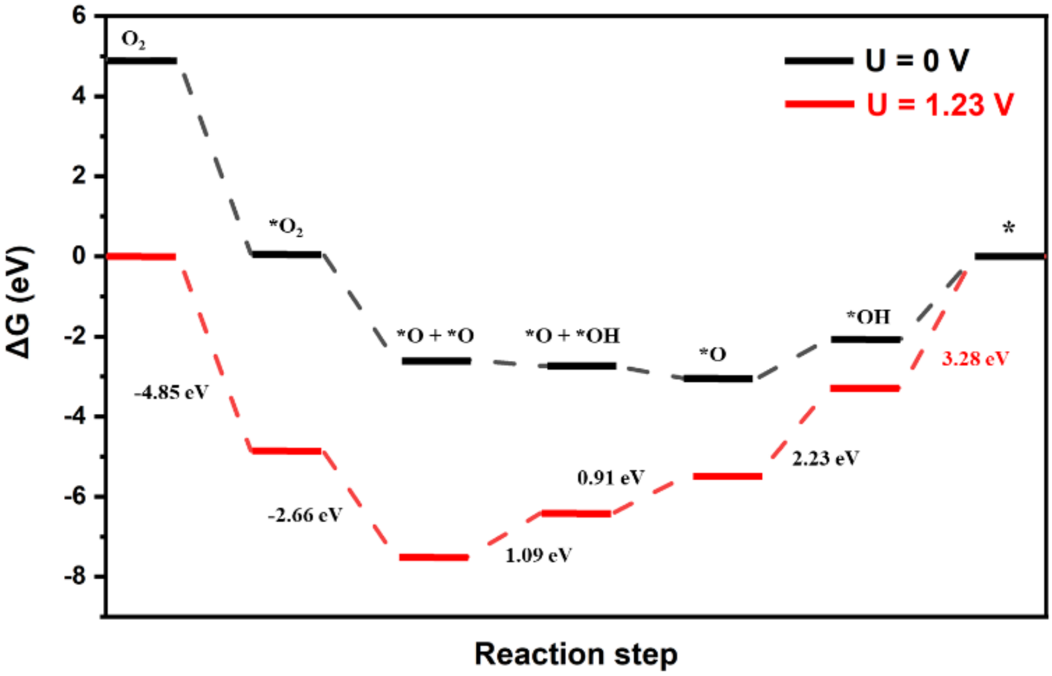

Figure S16. The free energy diagram of ORR on W atom in W SAs system.

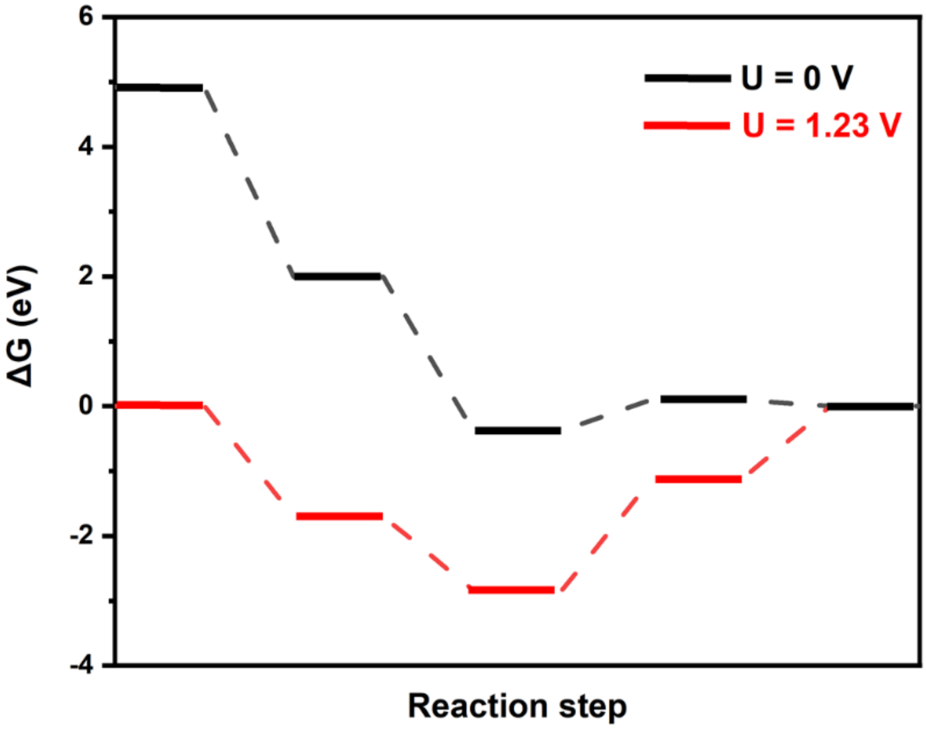

Figure S17. The free energy diagram of ORR on N2 atom in WNs system.

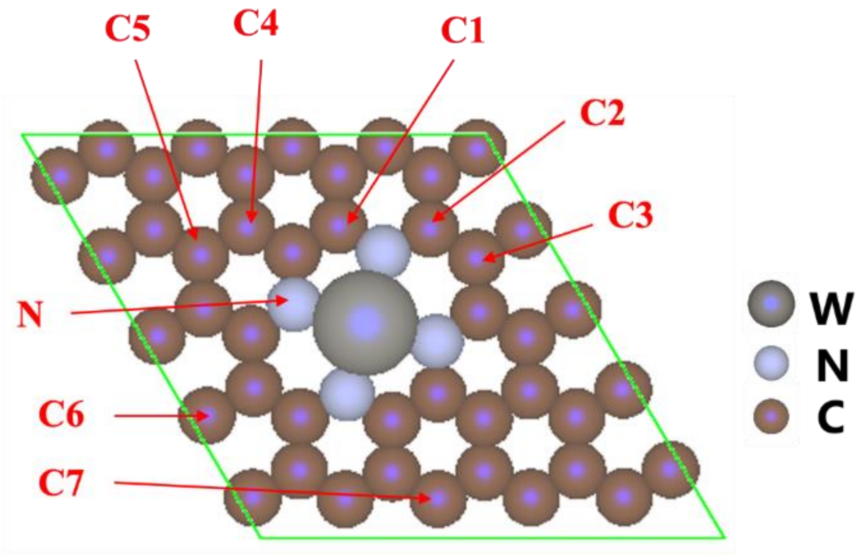

Figure S18. The structural models of W SAs system.

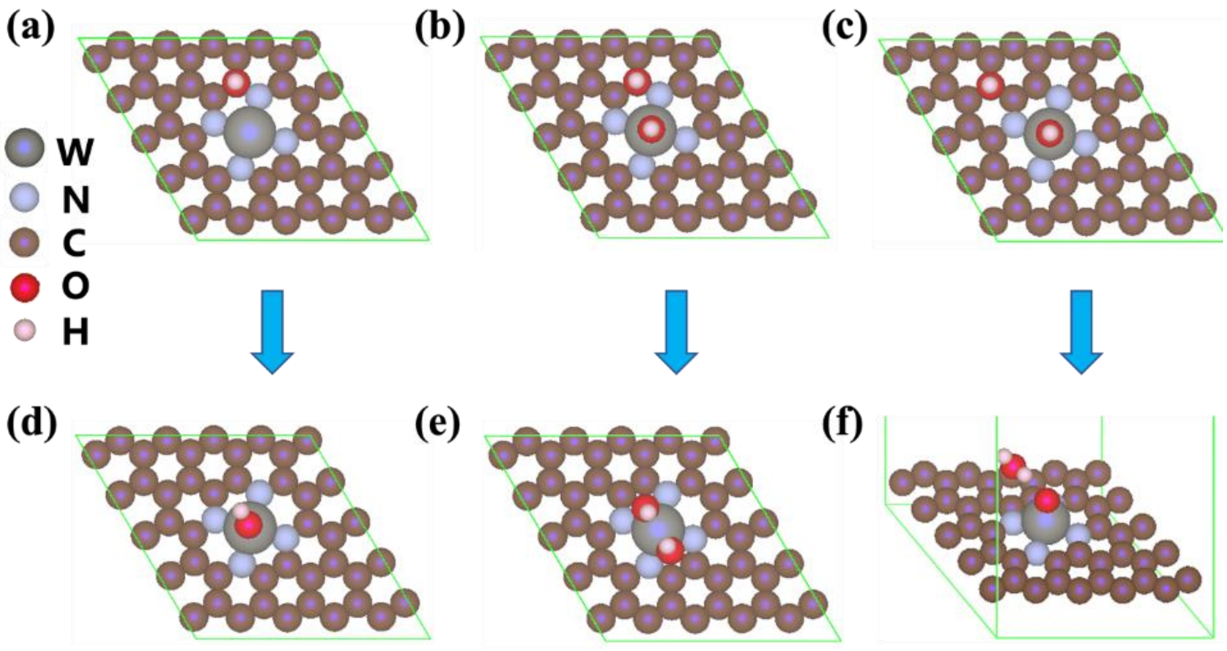

Figure S19. The initial structural models for: (a) C1, (b) C1 with W passivation and (c) C4 with W passivation in W SAs system before calculation. (d), (e) and (f) were the corresponding final structural models of (a), (b) and (c), respectively, after calculation.

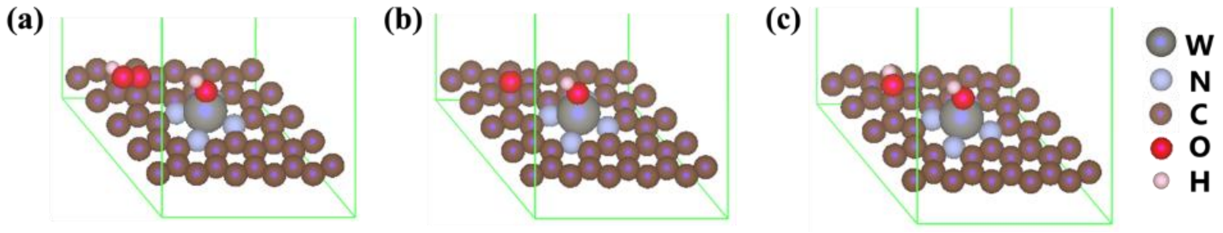

Figure S20. The structural models of (a) *OOH adsorption, (b) *O adsorption, and (c) *OH adsorption on C5 atom in W SAs system.

Table S1. BET surface areas for all catalysts

	W SAs/WNNC-5	W SAs/WNNC-10	WNNC-20	WNNC-40
BET surface areas (m^2g^{-1})	1115.1	1105.3	891.2	568.0

Table S2. Comparison table of catalytic performance

Catalysts	E_{onset}/ V vs RHE	Stability	Reference
**Ir-SAC**	0.81	Nearly unchanged after 5000 cycles	[31]
**Ir-N/C**	0.923	97% retention in current density after 10000 seconds	[32]
**Co-N_4**	0.876	NA	[33]
**Mn-N-C900**	0.98	the E1/2 value decreases about 16 mV after 10000 cycles	[34]
**WN@g-C_3N_4**	0.92	7% attenuation of catalytic activity after 55000 s	[35]
**W-N-C/700**	0.848	10.5% lost in current density	[36]
**W SAs/WNNC-10**	0.88		This work
**WNNC-20**	0.87		This work
**WNNC-40**	0.83		This work
**W SAs/WNNC-5**	0.89		This work

Table S3. Performance of Zn-air batteries reported in literature.

Materials	Battery Voltage (V)	Cycling Time (h)	Reference
**W SAs/WNNC-5**	1.34 (solid)	**13**	**This work**
Fe_2N@carbon	1.43 (solid)	14.5	[37]
N-GCNT/FeCo	1.25 (solid)	12	[38]
Co_3O_4 N-rGO	1.31 (solid)	2	[39]
HCA-Co	1.39 (solid)	41	[40]
NCNF film	1.256 (solid)	6	[41]
Pt/C-Ir/C	1.39 (solid)	13.5	[42]

Table S4. Performance of Al-air batteries reported in literature.

Catalyst	Battery Voltage (V)	Discharge Time (h)	Reference
**W SAs/WNNC-5**	**2.01 (solid)**	**≈ 4.3**	**This work**
Co-N/CNT	1.65 (liquid)	≈ 7	[43]
A typical porous material	≈ 1.3 (solid)	≈ 7	[44]
Co_3O_4-CeO_2/KB	1.27 (liquid)	≈ 10	[45]
Fe-N-C	1.35 (liquid)	≈ 1.4	[46]
Ag-CNT	≈ 1.4 (solid)	none	[47]
HCA-Co	1.97 (solid)	≈ 8.5	[40]

Table S5. The adsorption energy of *OH.

	C5	C6	C7
Adsorption energy (eV)	-2.12	-2.47	-2.44

Reference

[1] Kresse G, Furthmüller J. Efficient iterative schemes for ab initio total-energy calculations using a plane-wave basis set[J]. Physical review B, 1996, 54(16): 11169.

[2] Kresse G, Joubert D. From ultrasoft pseudopotentials to the projector augmented-wave method[J]. Physical review b, 1999, 59(3): 1758.

[3] Blöchl P E. Projector augmented-wave method[J]. Physical review B, 1994, 50(24): 17953.

[4] Perdew J P, Burke K, Ernzerhof M. Generalized gradient approximation made simple[J]. Physical review letters, 1996, 77(18): 3865.

[5] Grimme S, Antony J, Ehrlich S, et al. A consistent and accurate ab initio parametrization of density functional dispersion correction (DFT-D) for the 94 elements H-Pu[J]. The Journal of chemical physics, 2010, 132(15): 154104.

[6] Grimme S, Ehrlich S, Goerigk L. Effect of the damping function in dispersion corrected density functional theory[J]. Journal of computational chemistry, 2011, 32(7): 1456-1465.

[7] Monkhorst H J, Pack J D. Special points for Brillouin-zone integrations[J]. Physical review B, 1976, 13(12): 5188.

[8] Nørskov J K, Rossmeisl J, Logadottir A, et al. Origin of the overpotential for oxygen reduction at a fuel-cell cathode[J]. The Journal of Physical Chemistry B, 2004, 108(46): 17886-17892.

[9] Li L, Huang R, Cao X, et al. Computational screening of efficient graphene-supported transition metal single atom catalysts toward the oxygen reduction reaction[J]. Journal of Materials Chemistry A, 2020, 8(37): 19319-19327.
